# A potent cadmium bioaccumulating *Enterobacter cloacae* strain displays phytobeneficial property in Cd-exposed rice seedlings

**DOI:** 10.1016/j.crmicr.2021.100101

**Published:** 2021-12-18

**Authors:** Antara Ghosh, Krishnendu Pramanik, Shatabda Bhattacharya, Sayanta Mondal, Sudip Kumar Ghosh, Tushar Kanti Maiti

**Affiliations:** a Microbiology Laboratory, Department of Botany, The University of Burdwan, Purba Bardhaman, West Bengal 713104, India; b Mycology and Plant Pathology Laboratory, Department of Botany, Visva-Bharati, Siksha Bhavana, Birbhum, Santiniketan, West Bengal 731235, India; c Department of Materials Science and Engineering, Seoul National University, Seoul 08826, South Korea

**Keywords:** Cadmium resistant PGPR, Enterobacter cloacae, ACC deaminase, Antioxidant enzymes, Bioaccumulation

## Abstract

In agricultural soil, cadmium (Cd) pollution compromises soil health, reduces crop yield, and produces Cd-contaminated crops. Bio-based approaches are necessary as an eco-friendly and sustainable solution to mitigate Cd-polluted areas. A heavy metal-resistant rhizobacterial strain (AS10) has been isolated from a heavy metal-defiled rice field. The 16S rDNA sequence and MALDI-TOF MS analyses of ribosomal protein reveal its identity closely similar to *Enterobacter cloacae*. The strain was found to withstand up to 4000 μg/ml Cd^2+^, 3312 µg/ml Pb^2+^ and 1500 µg/ml As^3+^. The Cd^2+^ removal efficiency was recorded as high as 72.11% when grown in 4000 μg/ml Cd^2+^. The strain's Cd-accumulation efficiency was also apprehended by TEM-EDAX followed by XRD-XRF-FTIR analyses. Besides, the strain showed solubilization of inorganic phosphate, ACC deaminase activity, nitrogen fixation and IAA production ability. Added further, the strain, as an efficient bioinoculant, significantly improved rice plant growth at the seedling stage through Cd immobilization. It prevented the surge of stress ethylene and oxidative stress in rice seedlings, resulting in overall plant growth improvement. Hence, the strain AS10 as potent plant growth-promoting rhizobacteria (PGPR) may be beneficial, especially in heavy metal-contaminated crop fields.

## Highlights

• Cd-resistant and halotolerant PGPR *enterobacter cloacae* AS10 was isolated.
• AS10 showed IAA, HCN production, P-solubilization, N_2_ fixation, ACCD activity.
• AAS-TEM-EDAX-XRD-XRF-FTIR studies confirmed Cd bioaccumulation by AS10.
• AS10 reduced oxidative stress, Cd uptake and improved rice seedling growth *in vitro*.

## Graphical abstract

**Figure:**
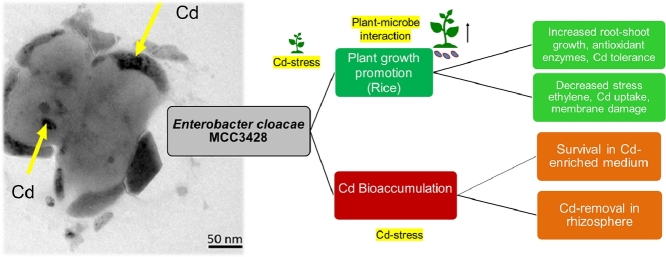

## 1 Introduction

Heavy metal toxicity and their accumulation in the food chain are increasing rapidly due to various anthropogenic activities resulting in a severe threat to ecosystems globally (Cuypers et al., 2010). Certain metals (Mn, Co, Fe, Zn, and Ni) act as micronutrients for organisms. In contrast, some heavy metals/metalloids such as Cd, Hg, Pb, As have no such role rather harmful to biological systems even at shallow doses (Balali-Mood et al., 2021).

Cd is amongst the top ten hazardous contaminants as enlisted by Agency for Toxic Substances and Disease Registry (ATSDR) and recognized as a potent carcinogen by the International Agency for Research on Cancer (IARC) (Joseph, 2009). Cd causes itai-itai in human (Aoshima, 2016), induces oxidative stress, impairs the normal functioning of the liver, lungs, kidneys, pancreas, testes, placenta, and bones (Cuypers et al., 2010). Cd is also detrimental to plant growth and crop productivity (Khanna et al., 2019). This toxic metal causes morphological, physicochemical changes and induced oxidative stresses in plants by affecting several organelles' structural properties and membrane functionality, triggering significant crop loss through inhibition of seed germination, lateral root formation, degradation of chlorophyll, and disturbance in stomatal conductance (Tran and Popova, 2013). As a result, reduction in photosynthetic rate, loss of nutrients (in the likes of P, Mg, Ca, and K), and disruption in the water transport system are observed (Tran and Popova, 2013). Furthermore, Cd greatly impacts soil microbial diversity because of high selection pressure in the particular niche (Yu et al., 2021). Microbial metabolism, including cell growth stages and differentiation processes, has been known to be adversely influenced after a certain threshold level (Khan et al., 2009).

India and a large part of South and South-East Asian countries consume rice as one of the staple foods (FAO, 2003). However, heavy metal and salinity are two major abiotic factors that are a big challenge in rice cultivation. Rice growing in Cd-contaminated fields can accumulate up to 22–24% of Cd in its biomass, whereas increased osmotic stress (due to elevated NaCl levels) drastically reduces rice productivity (Chunhabundit, 2016; Parida and Das, 2005). Though both stresses are responsible for poor rice growth and productivity, Cd stress is more hazardous than NaCl as it persists in rice grains that can directly enter the food chain.

So, the development of an efficient remediation measure is urgently required to take up the big challenge. Unlike traditional ways, bio-based approaches of soil-metal reduction are more promising as well as efficient, cost-effective, and eco-friendly for sustainable agricultural practices (Pal et al., 2019; Pramanik et al., 2021a). in this regard the use of heavy metal resistant plant growth-promoting rhizobacteria (PGPR) in soil bioremediation could be a useful approach. PGPR act as a biofertilizer (produces phytohormones, fixes nitrogen, solubilizes and mineralizes phosphate); bio-controlling agent (through siderophore, hydrogen cyanide (HCN) production), and stress-alleviator by producing 1-aminocyclopropane-1-carboxylic acid (ACC) deaminase. Bacterial ACC deaminase participates in maintaining the regular ACC pool in host plant cells and reduces stress ethylene (Pramanik et al., 2021b). The cumulative effect of these plant growth-promoting (PGP) traits augments plant growth in heavy metal-spiked fields.

Cd-resistant PGPR strains reported to date mainly falls under the following genera – *Pseudomonas* (Singh et al., 2015; Pramanik et al., 2021b), *Bacillus* (Singh et al., 2015; Ahmad et al., 2016), *Leifsonia* (Ahmad et al., 2016; Chen et al., 2016), *Klebsiella* (Ahmad et al., 2016; Pramanik et al., 2017; Mitra et al., 2018b), *Enterobacter* (Chen et al., 2016; Mitra et al., 2018a; Pramanik et al., 2018). Regardless, the search for efficient PGPR strains continues, which is imperative to develop effective bioinoculant for future use in contaminated agricultural fields.

This work aims to isolate a native PGPR strain through several rounds of screening, mainly based on multifarious PGP traits and heavy metal resistance. Furthermore, molecular identification of the strain was followed by a detailed characterization using a number of biophysical toolsas part of a mechanistic understanding of Cd-resistance. Finally, the investigation concluded with an assessment of the selected PGPR strain to explore its efficacy in alleviating stress-induced damages in rice seedlings.

## 2 Materials and methods

### 2.1 Site characterization, isolation, and preliminary characterization of Cd-resistant PGPR

The soil samples were acquired from rhizospheric soil of rice field of Nari, Burdwan district, West Bengal, India (GPS – 23°14′45′′N, 87°53′16′′E). The pH, salinity, phosphate, nitrate, and organic carbon of the samples were measured. Cd, Pb, and As content was monitored via atomic absorption spectrophotometer (AAS) following aqua-regia digestion (Hseu et al., 2002).

For isolation of desired PGPR, soil samples were serially diluted in sterile Millipore water, subsequently plated on 250 µg/ml Cd containing (CdCl_2_ as Cd source) Pikovskaya's agar media, and left for incubation for 24 h at 30 ± 2 °C. After the incubation period, Cd-resistant halo zone forming pure and distinct colonies were picked up and transferred to Davis Mingioli (DM) slants supplemented with 250 µg/ml Cd (sub-culturing interval-15 days) for further experiments. To screen a set of phytobeneficial or PGP traits such as indole-3-acetic (IAA) acid production, phosphate solubilization, nitrogen fixation ability, ACC deaminase activity, siderophore, and HCN production were assessed qualitatively (Pramanik et al., 2018). After primary screening based on various PGP traits with Cd added selective medium AS3, AS10, and AS11 were selected for future study. Furthermore, minimum inhibitory concentration (MIC) of heavy metal(loid)s (Andrews, 2001) were detected for the selected strains (AS3, AS10 and AS11) using different concentrations of Pb^2+^ [Pb(NO_3_)_2_], As^3+^ (NaAsO_2_) and Cd^2+^ (CdCl_2_). Further, the survival of the strain AS10 at such higher doses of Cd was confirmed by performing triphenyl tetrazolium chloride (TTC) test (Pandey and Bhatt, 2015) while growing the strain in the DM agar plate supplemented with 2250 μg/ml Cd. A Cd-sensitive strain - *Burkholderia* sp. P24 was taken as a control. The NaCl tolerance ability of all the three selected isolates was also inspected following the methods of Sarkar et al. (2018).

### 2.2 Quantification of phytobeneficial traits of the selected isolates

To assay ACC deaminase enzyme, overnight grown bacterial cultures were taken and centrifuged. The centrifuged pellet was washed with saline water, then suspended in the 260 μg/ml Cd supplemented nitrogen-free DM media and left at 32 ± 1 °C. The cells were harvested after 24 h by centrifugation at 8000 rpm (4 °C, 10 min). The amount of α-ketobutyrate produced by ACC degradation is measured by obtaining OD_540_ values followed by comparing the values with a calibration curve of α-ketobutyrate prepared earlier (Penrose and Glick, 2003). IAA production was carried out quantitatively using Salkowski's reagent (Glickmann and Dessaux, 1995). Quantitative estimation of solubilized phosphorus was measured using the ammonium molybdate method (Fiske and Subbarow, 1925). Nitrogenase activity was confirmed by using flame ionization detector (FID) equipped gas chromatography (GC, VARIAN CP3800)) following acetylene reduction assay (ARA) (Dilworth, 1966).

### 2.3 Polyphasic approach-based identification of AS10 strain

The identification of the selected strain AS10 (screened based on heavy metal/metalloid resistance property and presence of numerous important PGP traits) was carried out using a polyphasic approach which includes conventional phenotypic characterization (Benson, 1990), matrix-assisted laser desorption/ionization mass spectroscopy (MALDI-TOF MS)-based identification (Pulcrano et al., 2013), and 16S rDNA-based sequence homology described earlier (Ghosh et al., 2021). For drawing an evolutionary position on systematics, a distance-based phylogenetic tree was built using MEGA7 software by calculating the best-fitted model (The Biodesign Institute, Arizona, USA) (Kumar et al., 2016). The statistical significance of branch points was designed by 1000 bootstrap values (Felsenstein, 1985) and 16S rDNA sequence of the strain was deposited to NCBI database and the strain was deposited to National center for Microbial Resource (NCMR), National center for Cell Science (NCCS), Pune, India.

### 2.4 Effect of Cd on growth and IAA production efficacy of AS10 strain

The selected isolate AS10 was grown in media amended with different concentrations of Cd at 30 ± 2 °C and bacterial growth was measured spectrophotometrically at 540 nm till 72 h at 12 h interval. The IAA production was also measured with different Cd concentrations (at 530 nm) till 72 h at 12 h interval (Glickmann and Dessaux, 1995).

### 2.5 Monitoring Cd bioaccumulation studies of AS10 strain

Transmission electron microscope (TEM, JEOL-2011, 120 kV) equipped with energy dispersive X-Ray analysis (EDAX, Bruker X Flash 6130) was used to determine the intracellular Cd bioaccumulation by AS10. For this, the strain was simply grown in 2250 µg/ml Cd parallelly with a control (without Cd) for 24 h at 30 ± 2 °C. Bacterial cells were processed according to Chen et al. (2016). Similarly, grown bacterial culture was lyophilized X-ray diffraction (XRD, RICH SEIFERT-XRD 3000P, X-Ray Generator-Cu, 10 kV, 10 mA, wavelength 1.5418 Å) measurements (Arivalagan et al., 2014).

For Fourier transform infrared spectroscopy (FTIR) analysis, powdered bacterial samples as prepared during XRD (Deokar et al., 2013) were used in this measurement and tested with KBr pellets at room temperature by using FTIR spectrometer (NICOLET MAGNA IR 750). For X-Ray Fluorescence spectra (XRF, Bruker ARTAX - ELEMENT ANALYSER, Current-698µA, Time-300 S, Voltage-50 kV) analysis, lyophilized bacterial samples (AS10, and AS10+Cd) were used with XRF spectrometer following the method of Ghosh et al. (2021).

### 2.6 Cd removal efficiency of AS10

To determine the apt Cd removal from Cd amended media, AS10 was grown in DM broth media supplemented with 1000, 2000, 3000, and 4000 μg/ml of Cd. The cultures were grown up to 72 h in a rotary incubator shaker at 32 ± 1 °C. The bacterial growth and Cd removal efficiency were recorded at 12 h intervals. The supernatant of centrifuged (10,000 rpm for 15 min) bacterial culture was analyzed by AAS (PerkinElmer, USA) to quantify the residual Cd in the medium. Cd removal percentage was calculated through the following formula (Pandey and Bhatt, 2015):

$$Cd\;removal\;\%=\;\frac{IC-FC}{IC}\;\times 100$$

Where, IC is the initial concentration (μg/ml) of Cd in Cd-supplemented DM medium at zero time, and FC is the final concentration (μg/ml) of Cd in medium after12 h interval bacterial growth up to 72 h.

### 2.7 Plant growth experiment in the presence of Cd and AS10 strain

To study the effect of AS10 on rice seedlings, a Cd-sensitive rice cultivar Pratikshya (IET-15,191) was obtained from Krishi Vigyan Kendra, Indian Council of Agricultural Research (ICAR), Chinsurah, Hooghly, West Bengal, India. The EC50 (the effective concentration where 50% seed germination was inhibited) of rice seeds was assessed using CdCl_2_ in different concentration grades (0–400 μg/ml). Besides, germination% was also evaluated (Pramanik et al., 2017). For *in vitro* plant growth-promoting experiments, surface-sterilized seeds were imbibed in sterile Millipore water for 6 h. One-third of the imbibed seeds were inoculated with overnight grown bacterial suspension (OD_540_ = 0.01) (1 × 10^6^ CFU/ml) of AS10. The whole seed lot was divided into three different seedbeds (in triplicates). Among them, one seedbed had bacterized seeds, while the other two were devoid of them. The organization of seedbeds was similar for all the three sets *i.e.*, 200 ml glass beakers containing sterile absorbent cotton (approximately 2 cm in height), filled with 25 ml of sterile Hoagland's solution (Ahmad et al., 2016) and Whatman filter paper on top of it containing approximately 20 seeds. The whole setup (in triplicates) was designated as, control- [without Cd and AS10], EC50— [with Cd but without AS10], and EC50+AS10— [with Cd and AS10] was maintained in a plant growth chamber at 30 ± 2 °C in dark condition for three days. After that, all these sets were kept inside the same chamber with intermittent light (light/dark = 10 h/14 h) for 7 more days. After the completion of growth, different morphological growth parameters - shoot length, root length, fresh weight and dry weights of shoots (SFW and SDW), fresh weight and dry weights of roots (RFW and RDW), seedling vigor index (SVI) were determined (Pramanik et al., 2017). Among biochemical parameters total sugar (Dubois et al., 1956), total protein (Bradford, 1976), superoxide dismutase (SOD) (Fick and Qualset, 1975; Giannopolitis and Ries, 1977), catalase (CAT) (Aebi, 1984), proline (Bates, 1973), α-amylase, protease (Khan and Faust, 1967; Snell and Snell, 1971; Bradford, 1976), chlorophyll-a, chlorophyll-b, total chlorophyll (Arnon, 1949), and malonaldehyde (MDA) (Heath and Packer, 1968) contents were assessed. Furthermore, the stress ethylene was also quantified by establishing a fourth seedbed set up (with CdCl_2_ and CoCl_2_ but without AS10) in addition to the aforementioned three seedbeds set up and marked as EC50+CoCl_2_ (CoCl_2_ act as an inhibitor of ethylene biosynthesis) (Pramanik et al., 2017).

### 2.8 AS10 mediated reduction of Cd accumulation in rice seedlings

To determine Cd content in rice at the seedling stage, seedlings were grown in three different seedbed conditions (*i.e.*, control, EC50, and EC50+AS10) at 32 °C for 7 days and kept under intermittent light condition (light: dark = 10h: 14 h). Following acid digestion (HNO_3_ and HClO_4_ in 3:1 v/v ratio) of the dried seedlings, the samples were filtered for the AAS study (Yang et al., 2009) and XRF analysis to estimate the Cd content (Ghosh et al., 2021). Bioaccumulation of Cd was assessed by the modified method of (Ahmad et al., 2014) through the following formula:

$$Bioaccumulation\;factor=\frac{Cd\;concentraion\;in\;rice\;tissues}{Cd\;concentraion\;given\;in\;seed\;bed}$$

### 2.9 Statistical analysis

The tools used for statistical analysis are MS-EXCEL (version 2010) and Origin software (version 8.5). In this work, all the experiments were conducted in triplicates (Pramanik et al., 2018). The mean values are represented with the standard error (SE) (marked as ± in the tables) and also standard error bars are implemented in figure graphs. The significance of differences between the control and treated setswere evaluated by Student's *t*-test and differences between groups were calculated by one-way analysis of variance (ANOVA) test. Differences at *p* ≤ 0.05 were considered statistically significant.

## 3 Results and discussion

### 3.1 Site characterization, isolation, and preliminary characterization of Cd-resistant PGPR

Heavy metal contamination in crop fields drastically affects soil-dwelling microbes and constantly reduces the microflora diversity with its increasing concentration in soil. In this type of harsh soil conditions, heavy metal-resistant bacteria proved to be exceptional. Therefore, such contaminated agricultural fields were purposefully selected to screen out potent heavy metal resistant as well as growth-promoting rhizobacterial strains (Ahmad et al., 2016; Chen et al., 2016). Other than industrial sources, many other anthropogenic activities like agricultural practices, including various agrochemicals, also add a significant amount of heavy metal(loid)s in the field (Joseph, 2009; Mondal et al., 2021). The collected soil samples were found to be polluted with different hazardous heavy metal(loid)s (probably through the long-term use of agrochemicals) such as As, Cd, and Pb determined through the AAS study (Supplementary Table 1). This contaminated site was thoroughly explored for the isolation of heavy metal-resistant PGPR. Numerous root-associated soil bacteria having the dual functionality of heavy metal resistance and plant growth promotion have already been reported from such heavy metal contaminated sites (Ahmad et al., 2016; Chen et al., 2016; Sarathambal et al., 2017; Khanna et al., 2019; Pramanik et al., 2021c).

**Fig. 1:**
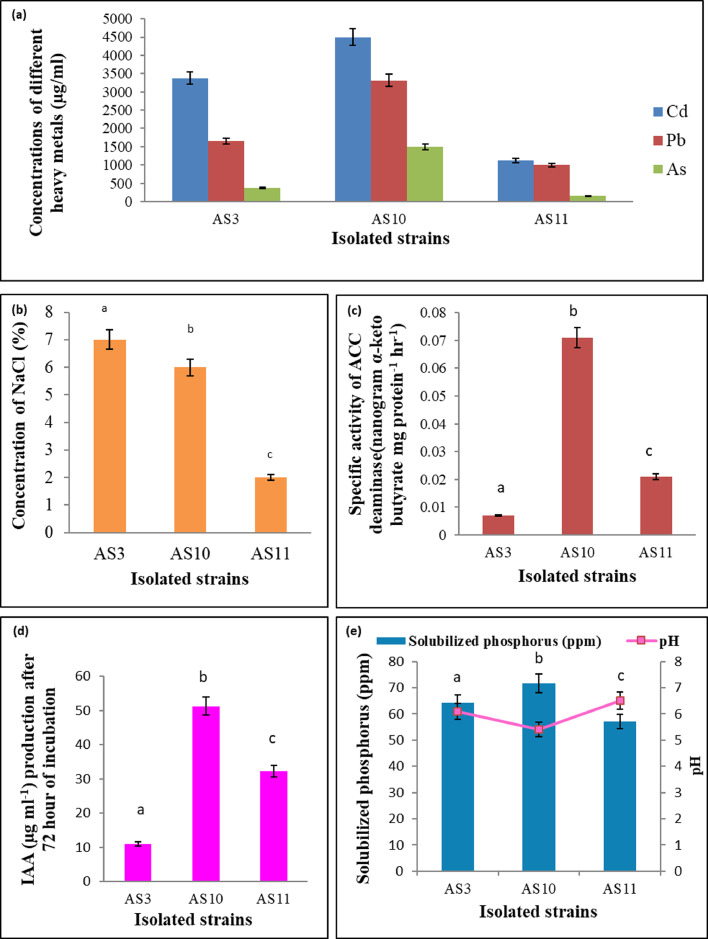
MIC determination and quantitative analysis of different PGP traits of selected heavy metal(loid) resistant isolates- (a) Heavy metal(loid) resistance,(b) Salt tolerance, (c) ACC deaminase activity,(d) IAA production, (e) Phosphate solubilization.

Three heavy metal(loid) resistant (Cd, Pb, and As) and halotolerant (NaCl) strains (AS3, AS10, and AS11) were isolated in this study which also showed several important PGP traits. These three strains were further scrutinized based on MIC values for those particular heavy metal(loid)s and tolerance levels in salt. AS3, AS10 and AS11 showed 7%, 6% and 2% salt tolerance limits respectively (Fig. 1b). But we selected AS10 strain for further study as it has better performance for other PGP characteristics and MIC of heavy metals compared to AS3 and AS11 strains. The strain AS10 showed the highest Cd, Pb, and As, tolerance (MIC values for Cd, Pb, and As were 4000, 3312, and 1500 µg/ml, respectively) (Fig.1a) and maximum NaCl tolerance (6%) (Fig.1b). To evade heavy metal and salt stress, some soil microbes have developed different resistance mechanisms while retaining several PGP traits, which ultimately serve them to tolerate, survive and grow successfully in such stressed conditions. The tetrazolium chloride (TTC) test is an effective and renowned test to check an organism's viability. Strain AS10 was found to withstand toxic doses of Cd (up to 4000 μg/ml). Therefore, it becomes legit to check for viability of the strain at such toxic Cd concentrations. The colorless and soluble TTC gives red colorization due to the formation of formazan in both Cd treated and untreated plates. The dehydrogenase activity is held responsible for such colorization and thus, formazan can be labeled as a metabolic indicator (Supplementary file Fig. 1). This result establishes the fact that AS10 remains metabolically active while living with high concentrations of Cd. The current finding is also sustained by earlier researchers (Pandey and Bhatt, 2015; Chen et al., 2016). Therefore, such PGPR can be utilized for better crop production in contaminated fields (Sengupta et al., 2015; Egamberdieva et al., 2019). The strain AS10 has greater ACC deaminase activity (Fig.1c), IAA producing capability (Fig.1d), and phosphate solubilization property (Fig.1e) when compared to other isolated strains. The strain also possesses better nitrogen-fixing ability (Supplementary file Table 2), ammonia, HCN, and siderophore producing activity among these three isolates (Supplementary file Table 3) as determined in qualitative and in quantitative experiments. Therefore, AS10 was selected unanimously for further experiments as it performed the best among the three isolates.

### 3.2 Quantification of phytobeneficial traits of the selected isolates

The phytohormone IAA is found associated with cell division, cell enlargement, root initiation, seed, and tuber germination. It also provides a degree of resistance against heavy metal stress that helps to stimulate rice seedling growth under Cd stress (Glick, 2012; Bhattacharyya and Jha, 2012). However, Cd disrupts IAA synthesis in plants by eliciting Cd-ROS-MAPK signaling, leading to senescence and cell death (Lin and Aarts, 2012). The strain AS10 is an efficient IAA producer (51.29 μg/ml) (Fig. 1d) that helps plant growth under Cd stress. It can promote root and shoot growth by increased nutrient acquisition (Chmielowska-Bąk et al., 2014). The IAA-producing ability of this strain was also positively correlated with the downregulation of Cd-ROS-MAPK signaling and the promotion of rice seedling growth under Cd stress (Treesubsuntorn et al.,2018).

AS10 can solubilize phosphate (71.7 ppm with a reduction in pH from 7 to 5.4) which augments plant biomass resulted in an increased crop yield in usual conditions as well as Cd-affected conditions. both under normal and stressed conditions (Naveed et al., 2014; Pal et al., 2019). The vital plant nutrient phosphorus is adequately present in soils but mainly inaccessible to plant due to its insoluble nature. Phosphorus is an imperative growth-limiting factor e in plants and is drastically reduced during stressed conditions. The phosphate solubilizing bacteria (PSB) improves the phosphate uptake in plants by converting it from insoluble to soluble forms (Zaidi et al., 2006). PSB improves phosphate availability to plants and alleviates Cd toxicity in contaminated fields (Park et al., 2010). The strain AS10 happens to be a Cd-resistant PSB and can promote plant growth in phosphate limiting conditions.

The Cd resistant AS10 strain exhibits ACC deaminase activity (Fig. 1c). It cleaves ACC (the immediate precursor in the ethylene biosynthesis pathway) into α-ketobutyrate and ammonia for the sake of reducing stress ethylene, a property that has been considered as a critical PGP trait (Sarkar et al., 2018; Li et al., 2019). The enhancement of root elongation even under Cd stress is probably related to the ACC deaminase activity of this strain (Belimov et al., 2005). The AS10 strain also exhibits N_2-_ fixing ability (Supplementary file Table 2) can be used as a biofertilizer (Bhattacharyya and Jha, 2012).

### 3.3 Identification of the selected Cd-resistant AS10 isolate

**Fig. 2:**
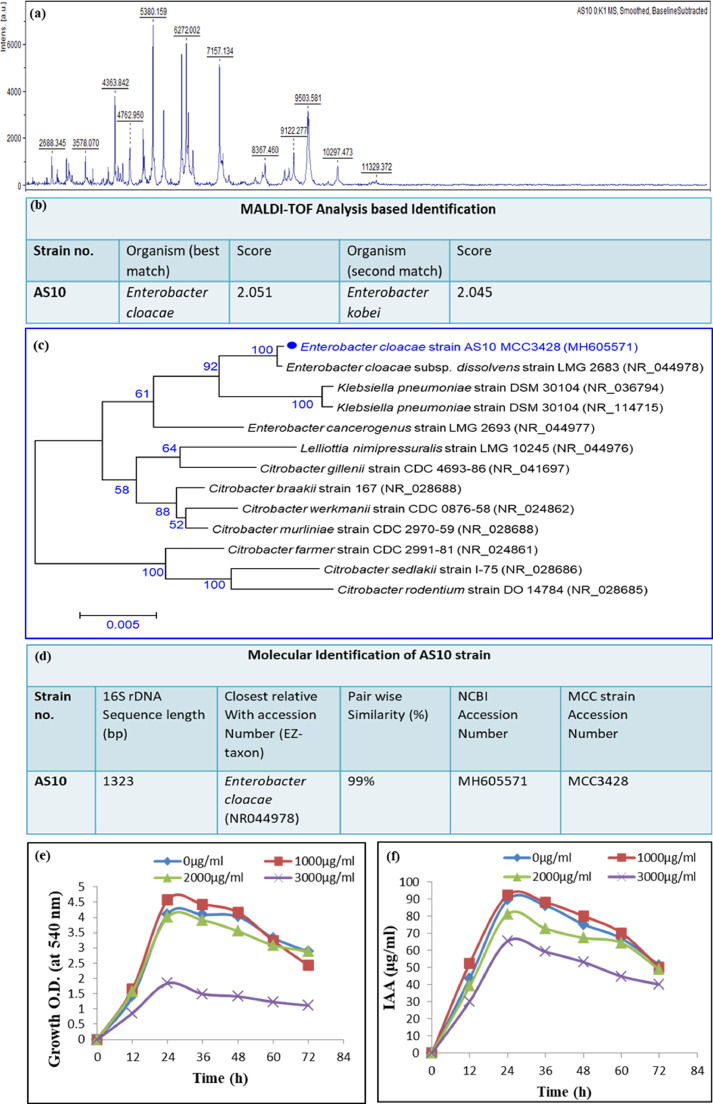
Identification and biochemical characterization of AS10 strain-(a) MALDI-TOF data of ribosomal protein, (b) MALDI-TOF analysis based Identification, (c) Phylogenetic tree of AS10, (d) 16 s rDNA sequence-based identification, (e) Effect of various Cd concentrations on bacterial growth, and (f) IAA production.

Primarily, the isolate AS10 was identified by MALDI-TOF MS (Fig.2a, b) and found that the selected organism was best matched with *Enterobacter cloacae*. Generally scoring more than 2 confirms its specific status. To confirm the identification further, phenotypic characterization and 16S rDNA sequence-based homology have also been performed. The morphological study shows that AS10 is a rod-shaped gram-negative strain (Supplementary file Table 4). The phylogenetic study revealed that AS10 showed 100% clustering with strain *Enterobacter cloacae* LMG 2683 (NR 044,978) (Fig. 2c). Thus, the species position of strain AS10 has been committed as *Enterobacter cloacae* based on polyphasic approach-based identification methods. The 16S rDNA sequencing data was accrued to NCBI database (sequence accession number is MH605571). Meanwhile, the strain was entrusted for global accession at Microbial Culture Collection (MCC), Pune, India, with the strain accession number MCC3428 (Fig.2d).

### 3.4 Impact of Cd stress on bacterial growth and IAA producing ability of AS10

In this segment, IAA production was quantitatively estimated under varied Cd concentrations along with the growth curve of AS10. From Fig. 2e and f, it was observed that the maximum growth along with maximum IAA production occurred in 1000 µg/ml Cd concentration at 24 h and decreased beyond this concentration with time. The result confirmed that the luxuriant bacterial growth accompanied by significant IAA production under Cd stress has a certain threshold level beyond which a steady decline in IAA production as well as in bacterial growth was observed.

### 3.5 Cd bioaccumulation by AS10 strain

**Fig. 3:**
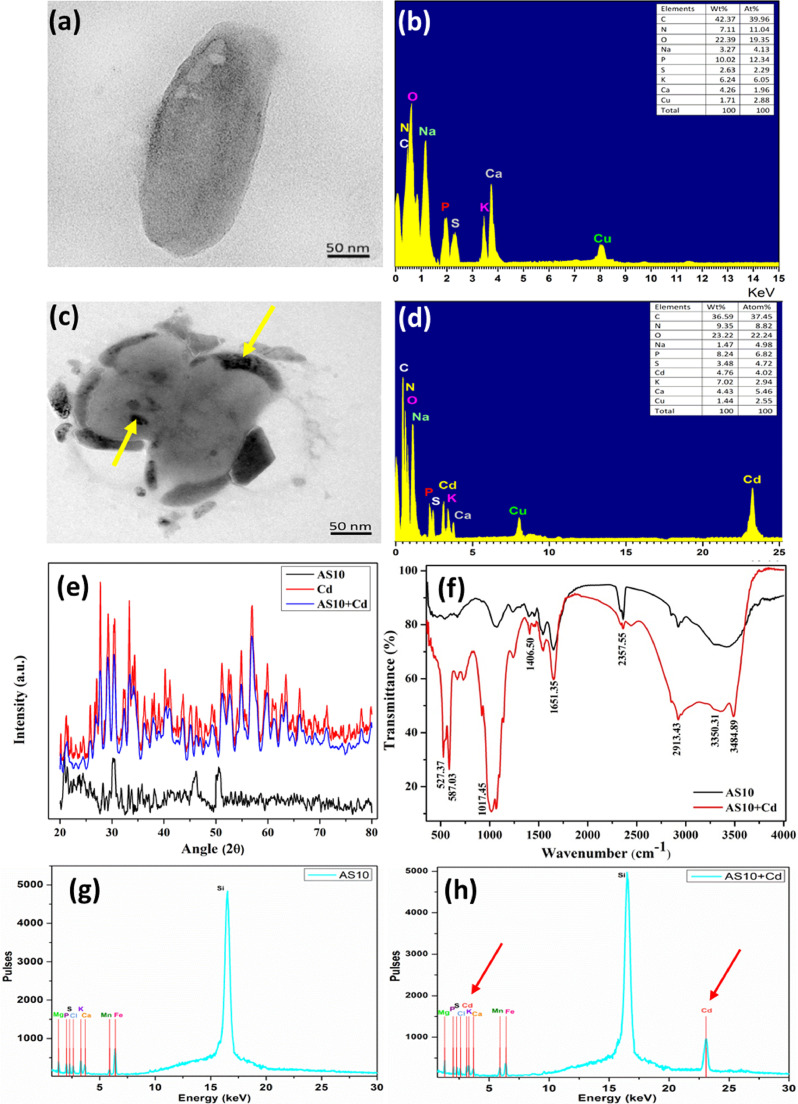
Cadmium bioaccumulation-related studies- (a, b) Cd untreated and (c, d) Cd treated cells of AS10 strain by TEM and EDAX studies. (e) XRD analysis of AS10; (f) FT-IR spectra analysis; (g) XRF spectra analysis of Cd untreated bacterial cells, (h) XRF of Cd treated bacterial cells.

The TEM study of strain AS10 clearly shows the intracellular Cd accumulation (electron-dense granules in Fig 3c) and it was further affirmed by EDAX study as the acute peak of Cd in treated cells, whereas, Cd untreated cells are devoid of electron-dense granules (Fig 3a) with no trace of Cd peak (Fig 3b). Prior studies have already established TEM-EDAX as affirmative methods for Cd bioaccumulation (Chen et al., 2016; Mitra et al., 2018). Thus, the strain might act as a barrier for plants shielding them from the Cd's toxic effects by accumulating Cd within itself. The relative absorption of Cd into the bacterial cell was measured by comparing Cd untreated and treated samples (Fig. 3e). The sharp diffraction peaks (in red color) showed the Cd level of the salt along with the crystalline phase of Cd. The black curve is indicative of Cd untreated bacterial samples where sharp diffraction peaks were absent. Whereas, a blue-colored peak renders the Cd treated profile of AS10. This study also serves as a confirmatory measure for Cd bioaccumulation.

To distinguish the interaction of Cd with various functional groups present in the cell surface, FTIR spectra analysis of Cd treated and untreated bacterial samples were carried out (Fig 3f). The transmission spectra at 3484.89 cm^−1^ and 3350.31 cm^−1^ are for O-H groups and N-H groups, respectively. The peak at 2913.43 cm^−1^ is attributed to C-H stretching bonds which belong to alkyl groups (Murthy et al., 2014). The spectra at 1651.35 and 1406.50 cm^−1^ are due to C=O and C-N groups. The value around 1017.45 cm^−1^ denotes phosphate groups. All the peaks are dipped at some point that further ensures the interaction of Cd with the bacterial cell. The results are positively correlated with previous studies with *Rhodococcus* sp. (Prasad et al., 2011) and *Bacillus aryabhattai* (Singh et al., 2016).

We have also carried out XRF analysis to detect various elemental components of Cd-treated and untreated bacterial samples (Fig. 3g and h) (Ghosh et al., 2021). In the untreated sample, Cd peak was not present (as well as other elemental peaks) whereas a prominent Cd peak was detected in treated samples. However, no peaks from other elements were detected in treated samples. Thus, this analysis further confirms the Cd absorption in bacterial cell pellets.

### 3.6 Cd removal efficiency of AS10

**Fig. 4:**
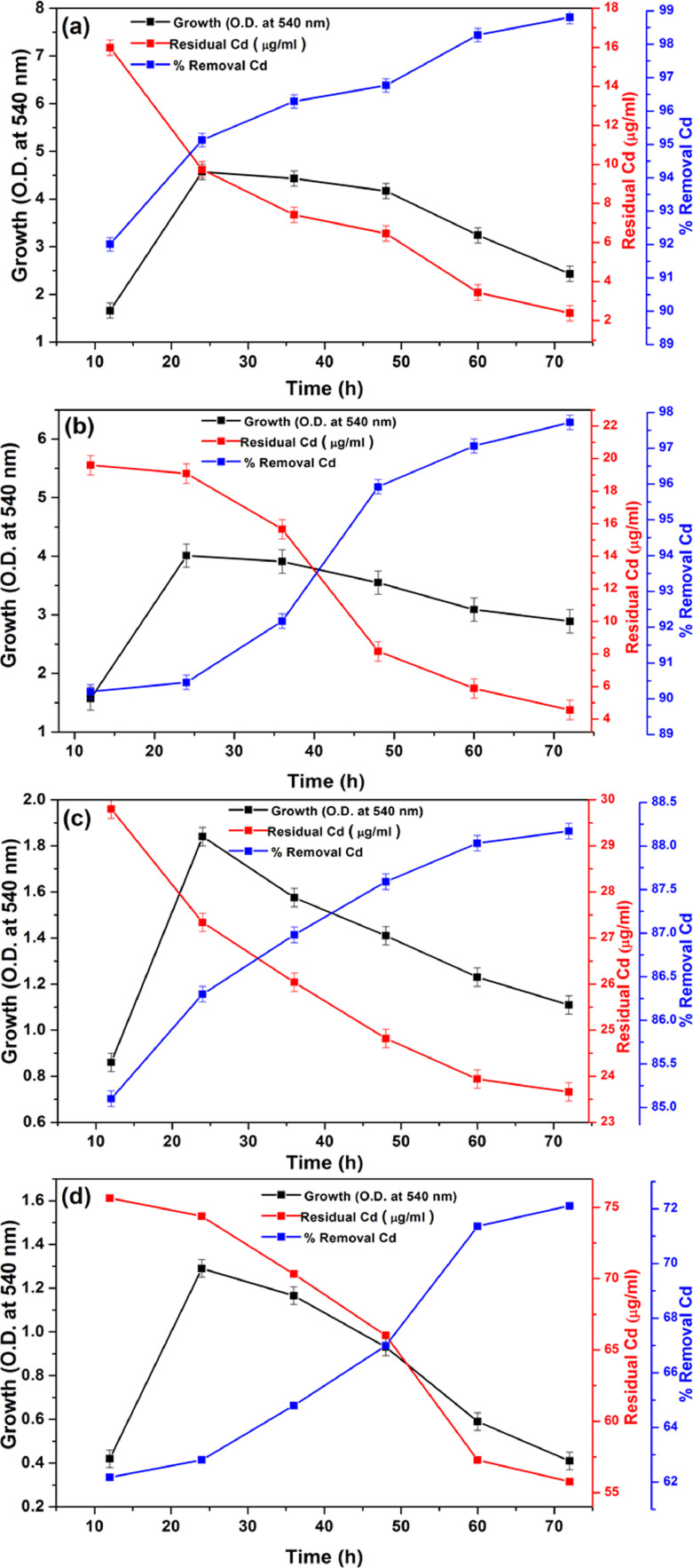
Determination of Cd removal efficiency of AS10 by AAS at various concentrations of Cd supplemented culture medium – (a) 1000 μg/ml Cd, (b) 2000 μg/ml Cd, (c) 3000 μg/ml Cd and, (d) 4000 μg/ml Cd.

The MIC value for Cd of PGP isolates AS10 was found to be 4000 μg/ml. However, the bacterial strain grew richly in 1000, 2000, 3000, and 4000 μg/ml Cd- supplemented DM broth. This study proves that the strain AS10 grows as a Cd- resistant strain under such high Cd-concentration and has Cd removal efficiency from the Cd-supplemented culture medium. The maximum Cd^2+^ abstraction detected by AAS in 1000, 2000, 3000 and 4000 μg/ml in Cd^2+^supplemented DM medium were 98.81%, 97.72%, 88.17% and 72.11%, respectively each culture after 72 h incubation (Fig. 4a–d). It was also observed that the Cd-removal efficacy was higher in 1000 μg/ml than other aforementioned Cd-supplemented media concentrations by strain AS10. This study indicates that the Cd removal proficiency effectively increased with the rapid increase of incubation time in every case of Cd supplemented growth culture. The Cd-abstraction proficiency also enhanced with the bacterial biomass. The competence of Cd bioaccumulation of this PGPR strain has a positive prospect of Cd removal from Cd-contaminated agricultural fields and making it less available to plants, thereby alleviating Cd phytotoxicity effects and enhancing rice seedling growth under Cd stressed conditions. This result also positively correlated with the results of *Enterobacter* sp. (Abbas et al., 2014; Sharma et al., 2020).

### 3.7 Morphological improvements in rice seedlings upon AS10 bacterization

**Fig. 5:**
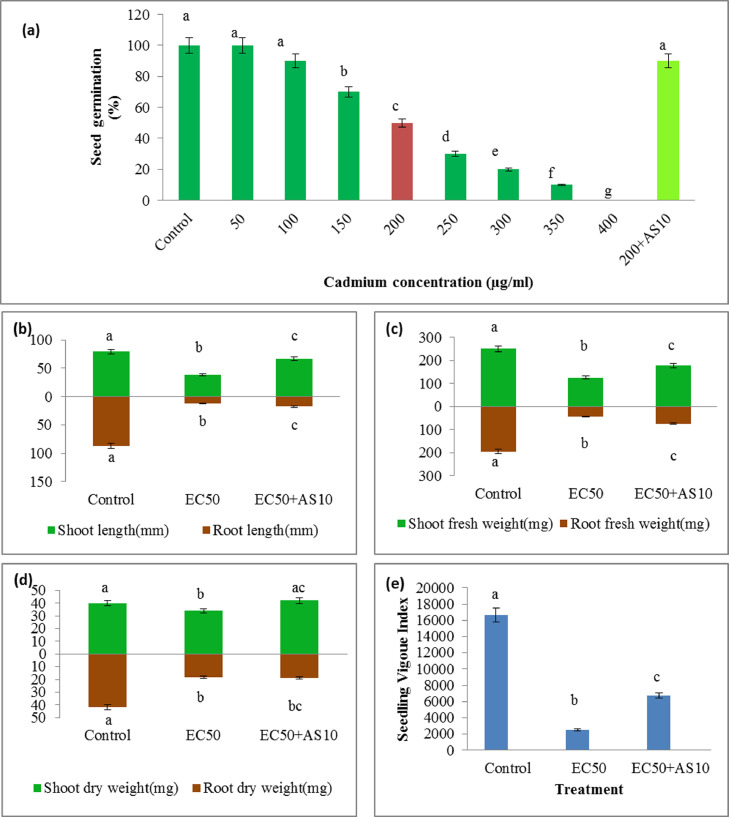
Effect of AS10 on various morphological parameters of rice seedlings under Cd stress-(a) seed germination (b) shoot-root length (c) shoot-root fresh weight (d) shoot-root dry weight and (e) seedling vigor index on rice seedlings under Cd stress.

A decreased germination percentage and reduced growth of the seedlings with increasing Cd concentration were observed (Fig. 5b). In this context, the effect of different PGP traits of AS10 under Cd stress positively affects rice seedlings' growth improvements performed *in vitro.* A visible impact of AS10 on the plant growth promotion of rice seedlings was observed in terms of different morphological and biochemical growth determinants.

AS10 significantly (*p* < 0.05) enhances seed germination% (up to 100%) (Fig. 5a). Besides, it significantly (*p* < 0.05) enhanced shoot length (> 1.69 times) (Fig. 5b), root length (>1.5times) (Fig. 5b), SFW (> 1.40 times) (Fig. 5c), RFW (>1.68 times) (Fig. 5c), SDW (> 1.23times) (Fig. 5d), RDW (1.05 times) (Fig. 5d), and SVI (Fig. 5e) when compared to Cd affected condition at EC50 (of rice cultivar) of 200 μg/ml. The improvements in shoot-root length, SFW, RFW, SDW, and RDW were probably linked to the plant growth-promoting effect of AS10 under Cd stress (Kamran et al., 2015; Khanna et al., 2019; Li et al., 2020). Increment of the shoot and root length in rice plants after application of *Serratia marcescens* (Kotoky et al., 2019) was observed under Cd stress. Moreover, the biomass increasing property (under Cd stress) of AS10 indicates the bioremediation capability of the isolate (Lin et al., 2016; Sharma et al., 2020). PGPR-mediated improvement in morphological growth indices under Cd stress has also been narrated earlier by other workers in this field (Ahmad et al., 2016; Li et al., 2019).

### 3.8 Biochemical improvements in rice seedlings upon AS10 bacterization

#### 3.8.1 Total sugar and total protein content

**Fig. 6:**
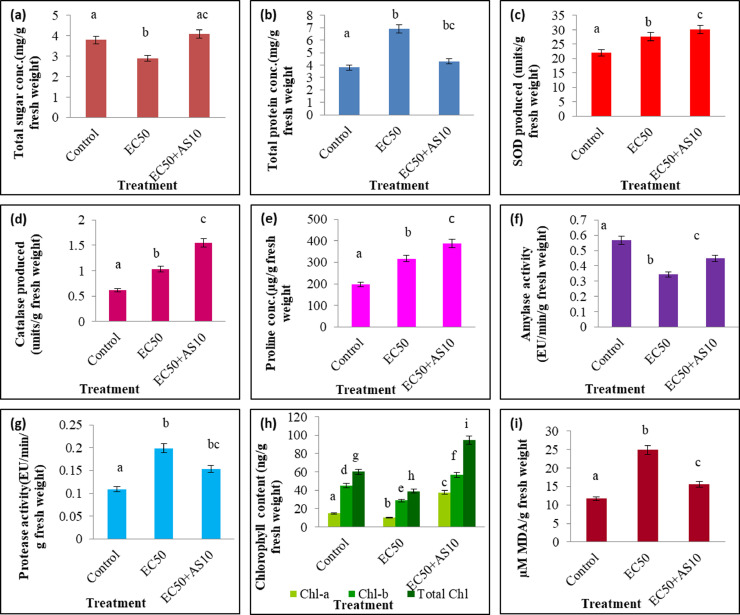
Effect of AS10 on various biochemical parameters of rice seedlings under Cd stress - (a) total sugar content, (b) total protein content, (c) SOD activity, (d) catalase activity, (e) proline content, (f) α-amylase activity, (g) protease activity, (h) chlorophyll (Chl-a, Chl-b and total Chl) contents, (i) MDA content.

Apart from morphological improvements, the strain AS10 was also found to regulate various biochemical parameters of rice seedlings under Cd stress. The amount of total sugar in bacterized seedlings was >1.40 fold compared to Cd-treated (EC50) seedlings (Fig. 6a). This significant (*p* < 0.05) rise in total sugar content in AS10 inoculated rice seedlings elucidates its part in ameliorating Cd toxicity. An increase in total sugar content is also known to provide osmoprotection and radical scavenging (Singh and Jha, 2016).On a contrary, a reduction in the total protein content on the application of AS10 was observed when compared to EC50. However, the values were significantly (*p* < 0.05) greater than that of the control set (Fig. 6b). Controlled proteolysis is required to maintain the protein homeostasis at the intracellular level, which further promotes plant growth under abiotic stresses (Kidric et al., 2014).

#### 3.8.2 Enzymatic antioxidant levels

Inoculating AS10 in rice seedlings significantly (*p* < 0.05) elevated SOD activity compared to EC50 (Fig. 6c). The toxic effect of heavy metal induces oxidative damage in plant cells. In response, plants synthesize antioxidant enzymes like SOD and catalase scavenge ROS to minimize oxidative damage and maintain redox homeostasis (Sharma et al., 2012). Cd-induced oxidative damage in plants is a common phenomenon as studied earlier (Cuypers et al., 2010). Catalase activity on AS10 treated seedlings was significantly (*p* < 0.05) upregulated when compared to both EC50 and control sets (Fig. 6d). A similar kind of catalase activity to counter Cd-induced oxidative damage has already been observed by many authors (Zhang et al., 2010; Islam et al., 2014).

#### 3.8.3 Non-enzymatic antioxidant levels

The stress buster amino acid proline is usually synthesized in response to various types of stresses (Hashem et al., 2016). Strain AS10 treated rice seedlings exhibited significantly (*p* < .05) higher proline content than both EC50 and control set (Fig. 6e). Increased proline content in rice seedlings is an ecological stress-induced adaptation found in plants that generally performs as a non-enzymatic antioxidative defense element (Hayat et al., 2012).

#### 3.8.4 α-amylase and protease activities

The α-amylase activity upon AS10 inoculation found to be significant (*p* < 0.05) thanEC50 set(Fig. 6f).This α-amylase activity might be connected with improved seed germination% (100%). PGPR-induced α-amylase activity upon Cd stress has been previously observed by various other researchers (Pramanik et al., 2018; Mitra et al., 2018). Enhanced α-amylase activity under abiotic stress could play a dynamic role in seed germination through hydrolysis of the storage polysaccharides (Ezraty et al., 2017). However, the protease activity was found to be decreased in AS10 treated seedlings compared to EC50 (Fig. 6g). The enzyme protease takes part in seed germination through the hydrolysis of storage proteins. So, the AS10 strain has a positive role in seed germination under Cd stress.

#### 3.8.5 Chlorophyll content

Cd-induced dysfunction in photosynthetic pigments (such as chl-a and chl-b) is a common phenomenon and is also perceived in our current study where the chlorophyll content of leaves of rice seedlings (EC50) was found to be reduced. However, bacterial inoculation (AS10) significantly (*p* < 0.05) improved chl-a, chl-b, and total chlorophyll content to overcome Cd toxicity (Fig. 6h). Therefore, it can be said that PGPR helps to curb stress-induced chlorophyll degradation and restore the normal photosynthetic activity of plants (Khanna et al., 2019; Houri et al., 2020). This result is positively correlated with plant growth promotion.

#### 3.8.6 Membrane lipid peroxidation

Malondialdehyde (MDA) is the end product of lipid peroxidation which is highly reactive and may also serve as a marker of stress-induced membrane damage. Higher MDA content in EC50 is due to peroxidation in membrane lipids, which indicates Cd-induced membrane damage in plants. The bacterial inoculation significantly (*p* < 0.05) decreases MDA content (Fig. 6i) thereby minimizing Cd-induced cell membrane damage. This result is corroborated with other previous works (Mitra et al., 2018; Pramanik et al., 2018).

### 3.9 AS10 mediated reduction in stress ethylene production of rice seedlings

**Fig. 7:**
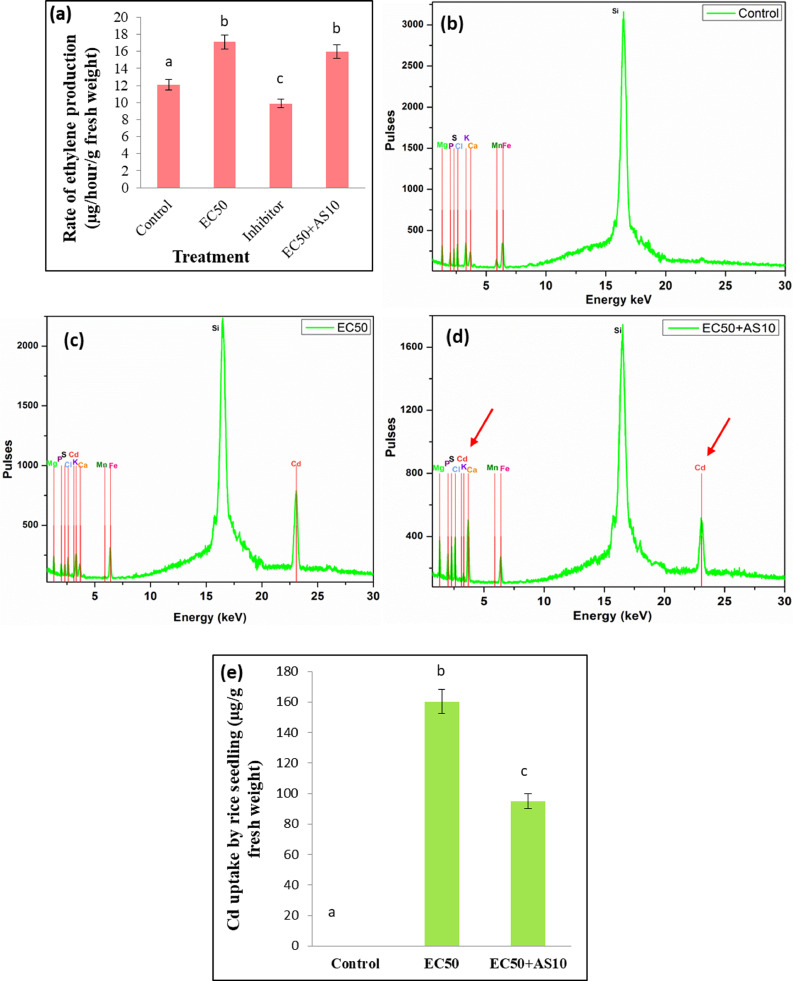
Stress ethylene production and reduction of Cd content in rice seedlings under the influence of AS10 -(a) Rate of stress ethylene production under Cd stress; (b) XRF of Cd untreated rice seedlings (control), (c) XRF of Cd treated rice seedlings (EC50), (d) XRF of Cd treated rice seedlings with bacterial inoculation(EC50+AS10), (e) Influence of AS10 on Cd uptake by rice seedlings.

Bacterization with AS10 showed reduced stress ethylene generation due to Cd stress (Fig.7a) compared to EC50, however, found increased compared to control and inhibitor (CoCl_2_) treated sets. This result is in accordance with the ACC deaminase (ACCD) activity of AS10 (Fig. 1c), which reduces unrestricted production of ethylene, thereby maintaining its correct proportion. This observation verifies that the ACC deaminase enzymatic activity of Cd resistant AS10 which is implicated in the hydrolysis of ACC to decreased abiotic stress-induced ethylene level hypothetically (Glick et al., 2005). It is reported that ethylene (higher level) has an important role in inducing apoptosis by Cd-induced hydrogen-peroxide (H_2_O_2_) production and Cd-resistant strain alleviates Cd phytotoxicity that conferred plant to tolerate Cd stress.

### 3.10 Role of AS10 in reducing Cd content from rice seedling

For elemental detection of Cd in plant samples, XRF analysis was carried out (Fig.7b–d). Definite Cd peaks were observed in EC50 and EC50+AS10 sets whereas no Cd peak was detected in the control set. However, Cd peaks were found to be less intense in the EC50+AS10 set due to efficient Cd sequestration by AS10. Besides, from AAS analysis of plant samples, it was found that Cd content was significantly (*p* < 0.05) reduced in seedling biomass after inoculation with AS10 strain (95 μg/g fresh weight) compared to EC50 under Cd stress (160.4 μg/g fresh weight). Thus, it can be concluded that the application of AS10 can reduce Cd uptake in rice seedlings as evident in our study (Fig. 7e). The result elucidates PGPR mediated reduction in Cd toxicity through minimizing Cd accumulation in plants. The Cd sequestration capability of Cd-resistant PGPR (like AS10) might serve as an essential tool in this regard. Intracellular Cd sequestration by *Enterobacter* sp. and plant growth promotion upon Cd stress has already been observed by different researchers (Chen et al., 2014; Li et al., 2019).

## 4 Conclusion

This study conclusively establishes the fact that the agrochemical-contaminated crop field instigates bacterial resistance towards heavy metals. The selected *Enterobacter cloacae* strain AS10 from the study site is a heavy metal-resistant halotolerant organism with several PGP traits with Cd- bioaccumulation ability. The strain promoted rice seedling growth through its multifarious PGP traits. The strain has a positive influence on different morphological, physiological, and biochemical growth parameters of plants that act together to reduce the toxic effects of Cd further. The strain promotes rice sapling growth and ultimate crop production under Cd stress conditions via various PGP parameters. Therefore, such strain can serve the dual purpose of bioremediation and bioaugmentation in Cd-contaminated sites in an environment-friendly manner. Hence, the strain should be further explored for Cd bioremediation and promote rice plant growth in Cd polluted agricultural land.

## Funding

This work is supported by the institutional fellowship (No.:2018/75), The University of Burdwan, West Bengal, India; Council for Scientific and Industrial Research (CSIR), India [Sanction letter no. 38(1469)/18/EMRII dated 04.04.2018]; University Grants Commission (UGC) – Dr. D. S. Kothari Fellowship [Award No. F.4–2/2006 (BSR)/BL/19–20/0072 dated 21.10.2019], New Delhi, India.

## CRediT authorship contribution statement

**Antara Ghosh:** Data curation, Formal analysis, Funding acquisition, Investigation, Methodology, Writing – original draft. **Krishnendu Pramanik:** Formal analysis, Funding acquisition, Investigation, Writing – review & editing. **Shatabda Bhattacharya:** Formal analysis, Investigation, Writing – review & editing. **Sayanta Mondal:** Formal analysis, Writing – review & editing. **Sudip Kumar Ghosh:** Formal analysis, Writing – review & editing. **Tushar Kanti Maiti:** Conceptualization, Project administration, Funding acquisition, Resources, Supervision, Validation.

## Declaration of Competing Interest

Authors declare that they have no conflict of interest.

## Appendix Supplementary materials

Image, application 1
