# Supplementary material for: A potent cadmium bioaccumulating Enterobacter cloacae strain displays phytobeneficial property in Cd-exposed rice seedlings
Source: Curr Res Microb Sci. 2021 Dec 18;3:100101. doi: 10.1016/j.crmicr.2021.100101 (PMC8724972; doi:10.1016/j.crmicr.2021.100101)
Supplement: Supplementary file 1 [file mmc1.docx]

**Supplementary file Table 1. Physicochemical characters of the soil sample of strain AS10**

| **Soil characters** | **Values** |
| --- | --- |
| pH | 7.1 |
| Salinity (psu) | 1 |
| Nitrate (µg/g dry wt.) | 1.4 |
| Phosphate (µg/g dry wt.) | 0.77 |
| Organic Carbon (%) | 1.1 |
| As (µg/g) | 32.14 |
| Cd (µg/g) | 0.62 |
| Pb (µg/g) | 15.94 |
| GPS location of  the study site | Nari P, Burdwan WB pin 713101 India  23°14’45” N 87°53’16” E |

**Supplementary file Table 2. Result of N_2_ fixation rate by ara of strain AS10**

| **SAMPLE** | **µg of N_2_ fixed hr^-1^** |
| --- | --- |
| AS 10 | 0.86 |

**Supplementary file Table 3. Plant growth promoting traits (qualitative) of the selected isolates**

| **PGP traits** | **Bacterial isolates** | | |
| --- | --- | --- | --- |
|  | AS3 | **AS10** | AS11 |
| ACC deaminase activity | **++** | **+++++** | **+++** |
| IAA production | **++** | **++++++** | **++++** |
| Phosphate solubilisation | **++++** | **+++++** | **+++** |
| Nitrogen fixation | **-** | **+++** | **-** |
| Siderophore production | **-** | **+++** | **-** |
| HCN production | **-** | **+++** | **-** |

‘+’ sign indicates positive result & ‘-’ sign indicates negative result.

Supplementary file Table 4. **Phenotypic characterization of AS10**

| **Colony Characteristics** | | **Results** |
| --- | --- | --- |
| 1 | Shape | Rod |
| 2 | Colour | white |
| 3 | Gram Nature | Gram (-) ve |

Supplementary file Figure 1. Viability test :(a)without Cd in medium, (b) Cd supplemented in medium.


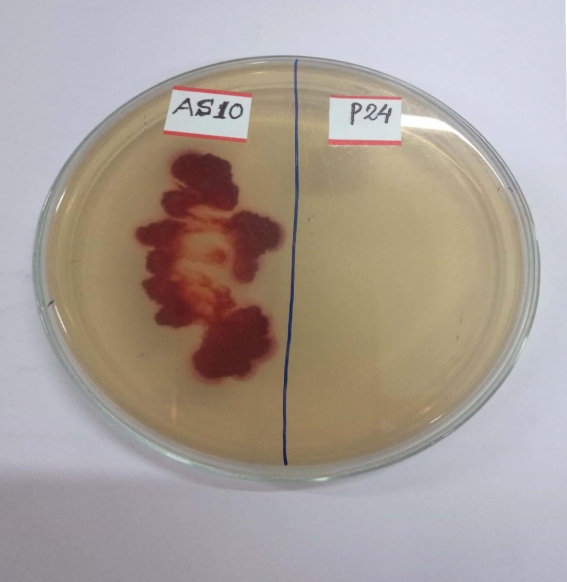

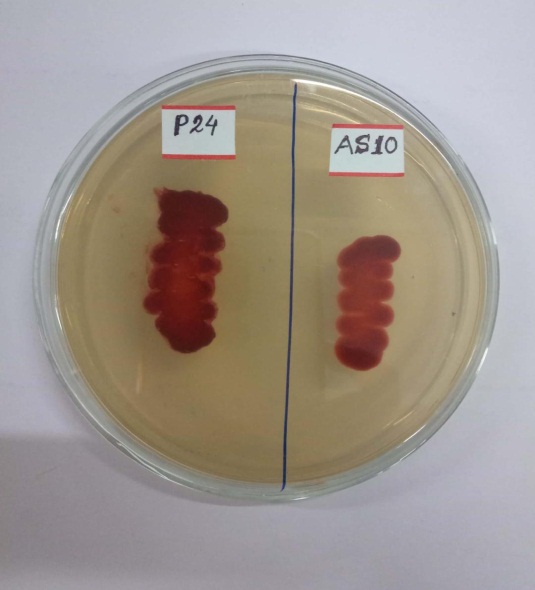


**(b)**

**(a)**

[Type a quote from the document or the summary of an interesting point. You can position the text box anywhere in the document. Use the Drawing Tools tab to change the formatting of the pull quote text box.]
